# Development, Acceptability and Engagement with the Mind Tutor: Lessons Learned from Testing a Novel Digital Application for First Year UK Undergraduate Student Wellbeing

**DOI:** 10.1007/s12646-025-00865-y

**Published:** 2025-10-25

**Authors:** Emma L. Davies, Natalie Wilde, Oliver Lennon, Alan Beck, Hazel Messenger, Kat Sergiou, Sarah E. Hennelly, Christian Ehrlich

**Affiliations:** 1 Centre for Psychological Research, Faculty of Health and Life Sciences, Oxford Brookes University, Headington, Oxford, OX3 0BP UK; 2 Syndeo Ltd, Syndeo House, 299 Ormeau Road, Belfast, Northern Ireland BT7 3GG UK; 3 London Metropolitan University, 166-220 Holloway Rd, London, N7 8DB UK; 4 Oxford Brookes Business School, Oxford Brookes University, Headington Oxford, OX3 0BP UK; 5 University of Birmingham, Birmingham, UK

**Keywords:** Chatbot, Undergraduate students, Wellbeing, Intervention development, Transitions

## Abstract

University student wellbeing has been declining in recent years, leading to increased demand for universities to provide support services. This paper aims to describe the development process of a novel chatbot enhanced app for student wellbeing, the Mind Tutor, and to evaluate the acceptability of this app. The paper also serves to provide multiple lessons learned from the development process and the testing of the app, which will be of use to other researchers in the field. Mind Tutor was developed with input from students and university service stakeholders. Findings from the development process suggested that the app should focus content on anxiety, mood, managing academic work, transitions and balance and relationships. Behaviour change techniques (BCTs) within the app were information provision, goal setting, mindfulness, skills/actions and reframing. Mind Tutor content relating to transitions appeared to be the most acceptable to students; however, engagement with the app was poor (two interactions per student). Although universities are keen to offer app-based wellbeing support to students, and students may like such tools, our study demonstrates that low engagement may impact the efficacy of such tools. Considerations for future research are discussed, including the practicality of randomised controlled trials, better integration with existing support services and integration with learning and teaching platforms. The paper also advocates for greater use of co-production methods, and the need to ensure appropriate face-to-face support is not superseded by digital technology.

## Introduction

### Context

University student mental health has been in decline in recent years (Johnston & Lis, 2022; Thorley, 2017). During the COVID-19 pandemic, lockdown conditions exacerbated the issue (Frampton & Smithies, 2021; Savage et al., 2020). Universities have also reported an overwhelming increase in demand for counselling services, leading to long waiting times for help (Thorley, 2017). Despite this increase in demand, evidence suggests only around 30% of students feel comfortable enough to seek help for mental health problems (Brown, 2018; Wilde et al., 2025). In a study by Eisenberg et al (2007), 84% of students currently experiencing anxiety and/or depression had not sought help, possibly because mental health problems are stigmatised (Aguirre Velasco et al., 2020; Gulliver et al., 2010). Further barriers to help seeking include feelings of self-reliance, lack of time and poor mental health literacy (Lui et al., 2022; Wilde et al., 2025).

First year undergraduates are at particular risk of poor mental health, in part, because the transition to university presents a significant period of change and uncertainty (Farrer et al., 2016; Thorley, 2017). When individuals start university, they often have to adapt to new, more self-directed methods of learning (Brown, 2018), which can lead to experiencing academic stress (Neves & Hewitt, 2021). Many students move away from home for the first time, losing their established social and emotional support networks (Macaskill, 2013). In addition, it is often the first time that young people have to manage their own finances, which has also been shown to have an impact on their mental health (Neves & Hewitt, 2021; Scanlon et al., 2007).

The use of self-help online tools may overcome barriers to help seeking for first year undergraduate students (and indeed students at other stages in their university studies). While not recommended to replace face-to-face interactions for more severe mental health problems (Dederichs et al., 2021), digital interventions can offer screening tools, activities to boost wellbeing and signpost for further support. However, systematic reviews suggest that more robust evidence is needed to evaluate the effectiveness of digital interventions for student mental health (Lattie et al., 2019).

### Chatbots and Mental Health

The current paper reports on the development, acceptability and engagement with the Mind Tutor, a novel chatbot enhanced app. Digital interventions increasingly use artificial intelligence (AI) tools. Chatbots are an AI tool that simulate conversation with a human and have been used in interventions as screening tools and to deliver therapies (Abd-alrazaq et al., 2019). For example, CBT principles have been delivered by a chatbot called the ‘Woebot’ to university students with anxiety and depression (Fitzpatrick et al., 2017). A small trial found this tool to be engaging and effective in reducing symptoms of depression when compared to a control group directed to web-based support (Fitzpatrick et al., 2017). Another chatbot called ‘Tess’ delivered CBT and motivational interviewing techniques and compared to a control group, participants who interacted with Tess had significantly lower depression, low mood and anxiety scores at two and four week follow up (Fulmer et al., 2018). While several chatbot apps are in existence, most research stems from outside of the UK context and does not solely focus on the unique needs of first year undergraduate students.

### Intervention Development

It is important to clearly define the assumptions and goals of an intervention and clarify the intended outcomes (Raymond et al., 2019). To this end, our development process involved specifying a logic model for the Mind Tutor. A logic model breaks the intervention into ‘inputs’, ‘activities’ or ‘processes’, immediate outputs and outcomes (short term and long term). Logic models have been applied in the past to community initiatives and domestic violence programmes (Hill & Thies, 2010; Kellogg Foundation, 2004) and other positive psychology interventions (Raymond et al., 2019). An additional important goal of intervention development is to accurately and precisely describe the content of the intervention. This includes the specification of behaviour change techniques (BCTs), which are defined as the active and replicable ingredients of behaviour change interventions, designed to alter the causal processes that regulate behaviour (Michie et al., 2013). In many cases the reporting of an intervention fails to adequately describe the BCTs that have been employed (Michie & Abraham, 2004; Prestwich et al., 2014). Therefore, it is hard to know exactly what works or what does not work for a given behaviour, thus limiting scientific advances in theory development and evidence-based practice, and making replication of successful interventions very challenging (Abraham & Michie, 2008). To that end, we describe the Mind Tutor content using the Behaviour Change Technique Taxonomy (Michie et al., 2013) within our logic model.

The Mind Tutor app was developed as a collaboration between academics, students and a software company. At the outset, the academic team engaged with students in focus groups and students in lectures and gained data from student support services while developing the intervention logic model. The behaviour change techniques were operationalised by academic experts in positive psychology and mindfulness. The software company then worked in ‘sprints’—focussed work periods—to build parts of the intervention, which were then discussed with the academic team. After a number of iterations, a version of the Mind Tutor was ready for testing in a randomised controlled trial (RCT). During the trial, a number of issues occurred within the app, for example content not appearing. A further version was then developed for testing in a second RCT. The results of both RCTs are reported elsewhere (Ehrlich et al. 2023). Protocols for the trials were registered on the Open Science Framework (Davies et al. 2022).

### Aims

The overall purpose of the current paper is to (1) describe the development of the Mind Tutor and (2) present evidence about its acceptability and how engaged students were with this novel app. This information will be useful to research teams aiming to develop and evaluate wellbeing tools for student populations.

## Methods

### Aim 1: Mind Tutor Development

During the intervention development process, the team collected data in focus groups with students and from stakeholder consultation (with wellbeing services, student support services and students). These data came from one university in the South East of the UK. There were 16,900 enrolled students in total at this university at the time, of which 83% were from the UK and 17% were international students and 41% identify as from Black, Asian or Minority Ethnic backgrounds. Acceptability and engagement data were collected from follow-up measures within the two RCTs.

#### Focus Groups

Online focus groups lasting 20–35 min were facilitated by a research assistant. Groups were designed to allow participants to discuss and clarify their views, prompted by the responses of other members (van Teijlingen & Pitchforth, 2006; see Box 1 for schedule).

**Box 1:** Focus group schedule

Focus group schedule **Introductory question** As you are aware we are here today to discuss what you think are the most important issues relating to studying at university and wellbeing relating for first year students To start the discussion, can you tell me what you think are the most exciting or interesting things about becoming a university student? **Key questions** *Studies* What concerns do you think students have in relation to approaching university study? How do you think most students feel about becoming independent learners? What are some of the barriers that students may face when trying to meet their study goals? *Wellbeing* What concerns do you think students have in relation to wellbeing? How do you think most students would respond if they or a friend were experiencing an issue that impacted their wellbeing? What do you think some of the barriers are to students seeking help for support with their wellbeing? *App related questions* Do any of currently use any app to help you either with your study goals or looking after your wellbeing? If there was an app that would aid you in completing your study goals, whilst also helping you to look after your wellbeing, are there any features that you think would be beneficial for that to have? If there was an app that included the features that you've talked about, just how long or how often do you think you would use it would it? **Summary/ending questions** Does anyone have anything they would like to add to the discussion?	

#### Participants

Participants were recruited from lectures and via student representatives. Inclusion criteria were that they needed to be enrolled undergraduate students who had completed the first year of studies. There were no exclusion criteria. In total, 14 students took part in five focus groups (four groups of three and one with two); There were four men and 10 women aged between 18 and 22. Five identified as White British, three as White—other, two as Asian, two as Black, one as multiple ethnicities and one did not provide their ethnicity. Discussions were detailed and rich; thus, this sample size was considered sufficient to provide information power—e.g. due to the specific focus of the research, a small number of participants were required (Malterud et al., 2015).

#### Analysis

Data were subjected to reflexive thematic analysis guided by Braun and Clarke’s six-step process (Braun & Clarke, 2019). A deductive approach was taken, guided by the aim of understanding what to include in the app.

#### Stakeholder Consultation (with Wellbeing and Student Support Services and Students)

In addition to focus groups with students, we consulted informally with wellbeing services, student support team members and second year students in a lecture. Wellbeing services are a central university function delivering counselling, disability and dyslexia support and welfare functions. Student support services are faculty based and aim to address initial requests for support relating to academic, personal and financial concerns. Both are delivered by non-academic members of staff. Information from wellbeing and student support services was gathered during unstructured face-to-face meetings with four representatives (one from wellbeing and three from student support services). In the meetings, we outlined the aim of our project and requested information about the most common reasons that students requested support during the previous three years. These data were pooled to give an overview of the most common reasons that student sought support.

**Fig. 1:**
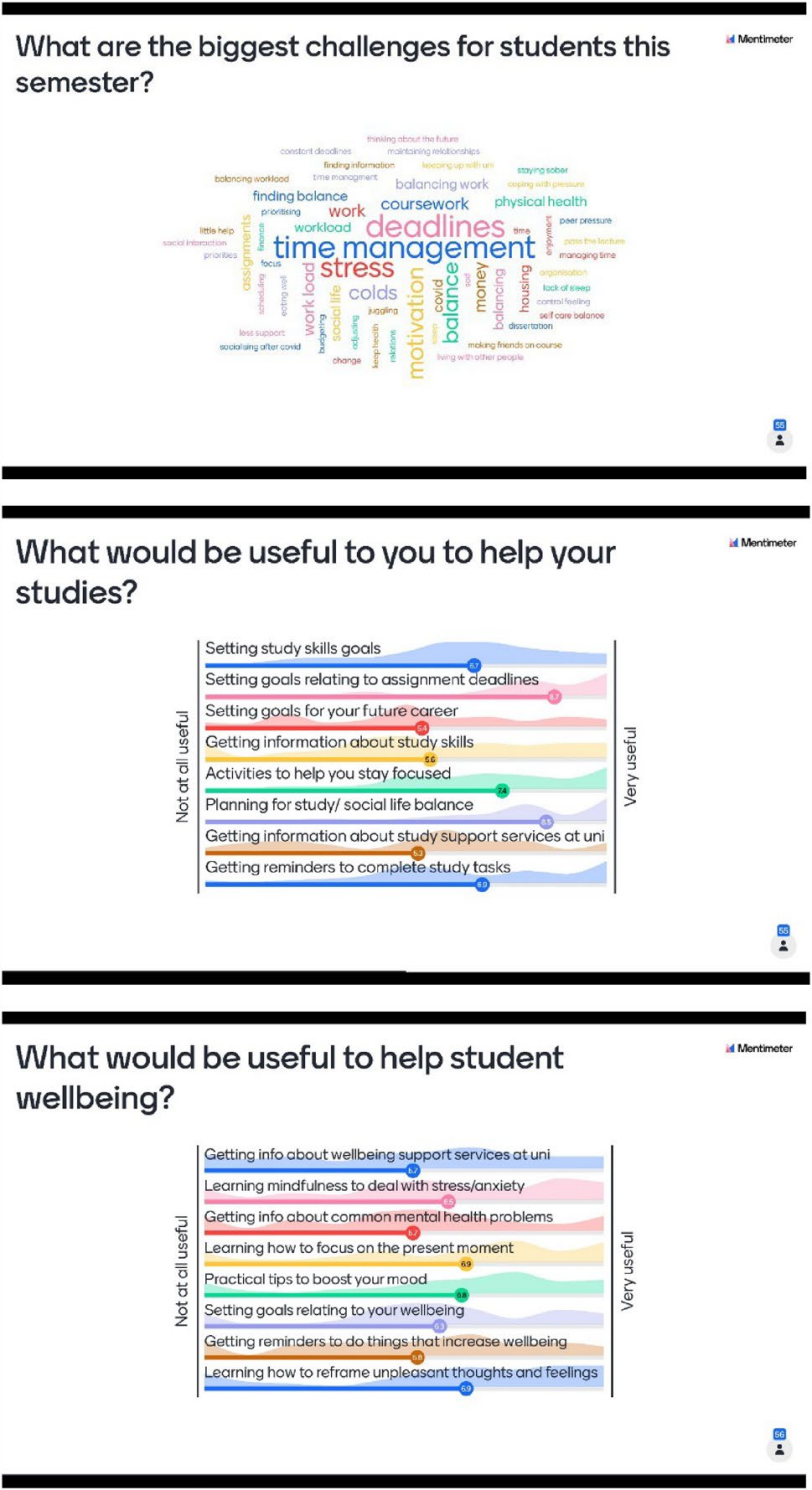
Results of Mentimeter class survey to gain feedback on Mind Tutor content. *Notes*: Words in wordcloud appear larger if they are more frequently mentioned. Waves in the background of the bottom two figures show the distribution of the data

Data from second year psychology students were gathered within a large lecture using the interactive teaching tool called Mentimeter. The rationale for consulting with second year students in this way was because they could reflect back on what would have been helpful to them during their first year of study. As this occurred at the start of semester, first year students would not have the experience or knowledge to know what they needed yet. Students viewed questions on screen and could use their mobile phones to provide anonymous responses. Three questions were posed. The first was an open question asking ‘What are the biggest challenges for students this semester?’ Two further questions asked students to rank priorities (from not at all useful to very useful) for the Mind Tutor in terms of support that would help with (1) studying and (2) wellbeing (see Fig. 1). Completing the questions was optional and anonymous, which means it is not possible to provide the range, mean and standard deviation for their age or to provide details of who did and did not take part. However, second year university students in the UK are typically aged between 19 and 21 years old.

### Aim 2: Acceptability and Engagement

In two randomised controlled trials, students completed a set of baseline wellbeing measures (including the Short Warwick Edinburgh Mental Wellbeing Scale (Stewart-Brown et al., 2009) and the Satisfaction with Life Scale (Diener et al., 1985)) and were then randomly allocated to either the Mind Tutor group or an inactive control. Full details of the trials are reported in Ehrlich et al., 2023. There were 177 students in the first trial (85 in the control group and 92 in the intervention group—6-week follow-up period) and 240 in the second (125 in each group—8-week follow-up period). There were different participants in each trial. The Mind Tutor group viewed a page with instructions on how to download the app from the Apple App Store or Google Play. There was no check to see if students had downloaded the app, nor were there instructions about how often to use it. However, those in the Mind Tutor group were followed up with an email prompt each week during the trials. Each reminder included the download link, as well as suggestions of what do in the app (e.g. ‘*How are you finding the Mind Tutor? We hope you’re finding what works for you. There are lots of features in the app. You can use it to set goals for studying. Don’t forget to use it this week’*).

#### Acceptability and Engagement Measures and Participants

Acceptability and engagement measures were collected in the follow-up data from both trials and were combined. All measures can be seen on the Open Science Framework (Davies et al., 2022). Both trials consisted of students from the same university as the focus groups, but with a small number of students from an additional UK based institution. Data were collected using an online survey software platform—Qualtrics. Participants were randomly allocated to either the intervention group or an inactive control group after completing baseline measures. Randomisation was through Qualtrics, which directed participants to a page to download the app, or a page explaining they were in the control group. In total, acceptability and engagement data were collected from 80 participants in the Mind Tutor group (16 men, 63 women, 1 non-binary;* M* age = 21.05, SD = 6.32) and 125 participants in the control group (48 men, 76 women, 1 non-binary; *M* age 20.16; SD = 4.52).

Acceptability: The following items from the baseline measures were analysed using descriptive statistics. The Mind Tutor group were asked: usefulness of topics (rated 1–5; higher being more useful); other topics they would find useful; to provide any further feedback on the app; and what else may have helped their wellbeing/studies over the trial period.

The control group were asked to answer hypothetically if they thought an app would have been beneficial to them; if they would have found the topics useful; other topics they would find useful; what would motivate them to use an app; and what else may have been useful to help their studies/wellbeing during the trial period. They had not accessed the Mind Tutor at all at that point.

Engagement: The Mind Tutor group were asked how often they had used the app; how long for; why they had not downloaded it if they had not; and what would have motivated them to use it more. In total, 80 participants provided data on engagement. Finally, analytics data about the usage of the app were gathered within the app. This allowed anonymous tracking of the daily interactions of each student with the app in terms of how many interactions and how long these interactions lasted.

## Results

### Aim (1) The Development Process

#### Focus Groups

Three main themes relating to student wellbeing and academic work were identified; coping with academic work, transitions to university life and other considerations for app development.

#### Coping with Academic Work

Participants discussed how managing their workload and being an independent learner were difficult. These were particularly challenging because of the difference between studying at school where teachers or parents might direct them, and the expectation at university that they would manage their own time.

*I think for a lot of people it’s quite a different environment, like the type of studies that go on like there’s lectures but mostly you just do work on your own and nobody really like, controls you (Woman, age 21)*

Deadlines seemed particularly stressful, as was the need to prioritise. As such, many the participants reported leaving work until the last minute, which lead to panic and feelings of stress. This had an impact on overall wellbeing.

*I think because in university there’s just so many things going on, so there’s submission of coursework, and then you have readings to catch up on, and then you have exams to study for, so I think what happens is you sort of don’t understand what to prioritize, so you would put the coursework deadlines, exams, university work on top of your wellbeing. (Woman, 21).*

#### Transitions to University Life

The transition to university life had positive as well as negative aspects and was particularly challenging for international students. Positive factors included having more freedom and the excitement of meeting new people. However, some mentioned being daunted by having to meet new people. Some felt they did not know their new friends well enough to talk to them about problems.

*Also, I think that, especially at the start of university, a lot of people might feel lonely, so they might not have somebody to turn to and trust. So, some people may focus too much on trying to please their peers and go out and have that unique experience, in inverted commas, that they think that they should be having (Woman, 20).*

Participants commonly talked about friends as a first line of support further highlighting the need to develop positive relationships at university. Linked to their need to develop new relationships, some students reported feeling a need to prioritise their social lives over their academic work. The challenge of being responsible for their finances and managing a balance between paid work and university work were also discussed.

*Another dimension is the work and life balance, sometimes the balance does tip over too much to life, so you know, they don’t really find a balance, so there’s also that (Man, 20).*

#### Other Considerations for App Development

Stigma about mental health problems and lack of knowledge about how to seek support were important identified issues that the use of an app could overcome. Many of the participants felt there were negative attitudes towards those who suffered mental health problems. They discussed feelings of shame, appearing weak, or embarrassed to discuss mental health.

*I would say, for example about mental health, it’s a little bit taboo, so people will feel judged when they talk about their wellbeing, and especially mental health wellbeing. I have lots of friends who can feel bad, but wouldn’t say anything because it’s still taboo to talk about mental health (Woman, age not provided).*

A lack of knowledge was identified about the types of support services available, how to access them, how much it would cost and what it was appropriate to contact them for. This information would then also be included as signposting within the Mind Tutor.

*Another thing I can add is that some students don’t know what they have an offer, and they might think they don’t know number one, what the services are, and second, they might think that it might cost a lot of money to get help. Then a second thing, especially during covid now, it has caused students to feel anxious about raising wellbeing related questions because they might currently not be prioritised? (Woman, 20).*

It is worth noting that some students made positive comments about the support that was available; however, they felt it might take a long time to be referred for support.

#### Stakeholder Consultation

##### Data from the Wellbeing Services

Following a meeting with a wellbeing team representative to outline the aims of the Mind Tutor study, data were supplied on the most common issues that students attended and presented with over the last three academic years (see Table 1). Anxiety was the most prevalent issue each year followed by mood related issues such as depression or anger. Academic issues were also in the top five each year, along with self and identity, and relationships.

**Table 1:** Top student wellbeing issues according to wellbeing and student support services

2018/2019		2019/2020		2020/2021	
Anxiety	309	Anxiety	313	Anxiety	291
Depression, anger & mood change or disorder	267	Depression, anger & mood change or disorder	294	Depression, anger & mood change or disorder	180
Academic	134	Self & identity	120	Self & identity	107
Relationships	98	Academic	90	Academic	64
Self & identity	92	Relationships	49	Relationships	46
Loss	73	Loss	48	Loss	34
Other mental health conditions	39	Abuse	36	Other mental health conditions	30
Physical health	39	Physical health	28	Abuse	26
Abuse	33	Other mental health conditions	26	Eating disorders	22
Eating disorders	21	Eating disorders	25	Physical health	18
Transitions	19	Transitions	13	Self-harm	12
Addictive behaviours	11				

##### Student Support Services

The student support service provided reasons that students most commonly turned to them for help. The most common reason that students sought support was for advice about their programme, including help selecting modules. Health and wellbeing was the second most common reason. This includes both mental and physical health, family/relationships as well as COVID-19 concerns in the 2019/2020 and 2020/2021 academic years. The third most common reason for students to contact the student support advisors was for help applying for mitigating circumstances relating to an assessment. Financial concerns were another common reason.

##### Student Feedback

In total, 55 students out of 75 in attendance chose to provide responses (response rate 73%). These students were particularly interested in goal setting around deadlines and planning for work–life balance. Activities to improve focus was another highly rated item. Learning to focus on the present moment and reframing unpleasant thoughts and feelings were rated highest for their use to help student wellbeing (see Fig. 1).

## Mind Tutor Content

The project team brought together all of the findings described above for discussion in a face-to-face meeting between the academic and industry partners. A logic model to describe the assumptions, features and intended outcomes of the Mind Tutor is shown in Table 2. Although a range of issues were raised as important, for practical reasons such as time, the five most highly rated topics were selected for inclusion, anxiety, mood, managing academic work, transitions and balance, and relationships. For each topic, we created a suite of interventions/activities matched to the topic. These were information provision, goal setting, mindfulness, skills/actions and reframing. A matrix to show how the topics and content fitted together is shown in Table 3.

**Table 2:** Mind tutor logic model

What is the problem	Key facets of the problem	Key audiences and point of entry to reach the audience	Problem theory	Intervention activities (behaviours that are undertaken within the app)	Short term outcomes to be measures in the study	Longer term anticipated outcomes
Poor mental health in students	Anxiety Low mood and depression Relationships Academic issues including completion of assessed and non-assessed work Transition to university life	First year students—key transition to independent study and transition to university	Increasing awareness of wellbeing issues addresses (and framing certain feelings as normal) lack of knowledge, and reduces stigma Frequent small doses of mindfulness may lead to increased ability to self-regulate attention and mood Goal setting allows tasks to be broken down into manageable chunks Short tasks can be practised to alleviate uncomfortable feelings in the short term and encourage healthy habits (for mental health and study habits)	Information about the issues, and signposting to support and reframing BCT 4.2 and 4.3 Mindfulness exercises for anxiety/mood Goal setting **BCT 1.1/1.3/1.4** Actions and skills **BCT 1.4/8.2**	**Subjective wellbeing** Self-efficacy Mindfulness Life satisfaction Mood	Lower levels of mental distress, lower levels of referral to wellbeing services Students stay on their course and progress with their studies more confidently
**Key assumptions**	Anxiety is the most prevalent mental health issue for students, followed by depression Students entering university in 2021 will have had disrupted education for two years	Students moving away from home and coming from school/college will face additional challenges relating to adjusting to independent life	Based on barriers and enablers to engaging in the behaviours within the app	Intervention exercises can be undertaken together, or as a suite of options depending on the individual need	Based on a minimum level of engagement during the trial	Based on continued use of the app

**Table 3:** Mind tutor matrix of topics and content

Topic/interventions	Anxiety	Mood	Transitions/balance	Academic	Relationships
Information provision	Micro-article about anxiety, what it is and what happens in the body	Micro-article about mood changes. Links to NHS sources on depression	Micro-article about starting university and having more freedom	Micro-article about managing time and understanding assignments	Micro-article about being connected to others and community
Goal setting—information and audio guided	Setting learning goals that focus on process and study skills rather than outcomes	Setting goals to ensure enjoyable activities are prioritised	Setting goals to trust your own decisions and become more independent	SMART goals and implementation intentions	Setting goals to reduce social comparison, setting goals for altruistic acts
Mindfulness—information and audio guided	Stop and breathe’ practices to settle the mind and body; brief ‘shake it off' movement to dispel anxiety	Short ‘stop and breathe’ practices and a three-step breathing space that aimed to help participants unhook from their current thoughts and feelings, and intermediate mood-focused ‘recognising emotions in the body’ practice	Five-to-seven-minute practices including a ‘body scan’ and ‘mindfulness of breathing practices that focused on noticing moment-by-moment changes and accepting the gap between expectations and experiences, and ‘beditation’ for taking a break to rest	Practices aimed to improve current moment focus on the tasks at hand using brief ‘stop and breathe’ and ‘breathe counting’ exercises	A foundational 'body scan' that focused on recognising we are all imperfect and complicated, and intermediate compassion practices focusing on unhooking from trying to solve someone and on softening our thoughts and feelings about another person
Skills and actions—suggestions for activities	Taking a short walk outside to quieten the mind	Ensuring sufficient sleep and exercise. Mood tracking	Trying new things on your own, making decisions	Breaking down a large task into smaller manageable chunks, taking breaks	Being assertive and having boundaries with other people—saying no
Reframing	Micro-article about how feeling anxious is normal, and can help us to perform at our best	Micro-article about how mood changes are normal and it is unrealistic to be happy all the time	Micro-article about how it is normal to feel unsure or uncomfortable when experiencing change	Micro-article about considering the broader purpose of studying, including transferrable skills	Micro-article about how feeling isolated might mean you need to reach how making new friends is hard for everyone

### Intervention Topics

1. *Anxiety *As one of the most common mental health problems faced by students, anxiety was important focus for the Mind Tutor (Thorley, 2017). Feedback from all of our stakeholders showed that this was an important concern. Existing research suggests that both generalised anxiety and social anxiety are concerns in the UK student population (Broglia et al., 2021).
2. *Mood *Our data showed that students commonly present to university counselling service with symptoms of depression (Broglia et al., 2021). Mood-related topics also relate to coping with feelings of low mood, anger, sadness and worry and enhancing positive emotions (Thorley, 2017). We used the word ‘mood’ rather than ‘depression’ to attempt to normalise mood fluctuations.
3. *Managing academic work *Academic distress is another common reason that students attend wellbeing services at universities in the UK (McKenzie et al., 2015). Working to deadlines, setting goals relating to assignments and understanding how to get support are key issues in enabling students to manage their academic work. In particular, our data from students in the lecture scenario highlighted a need to give students further support with this topic.
4. *Transitions/balance *Becoming independent in work and life, balancing work and social life and feeling a sense of autonomy are further challenges that students face (Thorley, 2017). Our data show that while this transition is an exciting time for students, there are uncertainties that may lead to a loss of confidence or distress. Balance is a key aspect here as shown within our focus groups and lecture survey.
5. *Relationships—creating and maintaining connections with others *Connecting with other people is vital. When students move away from home, they must develop new friendships as well as stay connected to others at home and elsewhere. As friends may be the first people they turn to when they are feeling worried about studying or their wellbeing, then maintaining new connections is also important.

### Behaviour Change Techniques

Within the Mind Tutor, participants interacted with a chatbot, which identified which of the five topics they need help with. Once this is identified, the Mind Tutor directs the participant to receive one of five interventions. Participants may then complete a further intervention, or all possible interventions within that topic. They may also go back to the main menu and start looking at another topic. The interventions consist of the following behaviour change techniques (BCTs) and/or tools. Table 2 shows the BCTs within the Mind Tutor. BCT numbers refer to the Behaviour Change Technique Taxonomy version 1 (Michie et al., 2013).

1. Information provision (BCT 4.2)

A recent study identified low levels of knowledge about mental health problems within a UK student sample (Wilde et al 2025). Thus, the Mind Tutor included information about the five topics with the aim of increasing knowledge of the issues as a starting point.

2. Goal setting (BCT 1.1/1.3/1.4)

Examples of goal-setting techniques in Mind Tutor were learning goals (Grant & Dweck, 2003), implementation intentions (Gollwitzer, 1999) as well as exercises based on the goal-striving reasons framework (Author). All three of those interventions have been shown to significantly related to people’s wellbeing. Hence, they have been selected in a goal-oriented environment such as student learning/academic performance.

3. Mindfulness (BCT 11.2).

Low-dose mindfulness is effective in improving psychological resilience and coping and in reducing exam stress in undergraduate populations (Galante et al., 2021; Loucks et al., 2021). Mindfulness activities were delivered by audio recorded by a trained mindfulness teacher. Activities included stop and breathe practices to settle the mind and body, body scan exercises to notice how the body feels and mindful breathing activities to notice moment-by-moment changes.

4. Skills and actions (BCT 1.4/8.2)

Taking a small step towards a larger goal, or developing a new coping strategy to deal with feelings of unease can alleviate feelings of worry in the short term (Jacob et al., 2022). Thus, the Mind Tutor included a number of suggested skills and actions for participants to try out. These include writing down feelings, taking a break and going outside, taking exercise and making a study calendar.

5. Reframing (BCT 4.3)

Reframing is a technique to aid with coping in stressful situations. Students may encounter numerous new and unfamiliar stressors when they enter university. It is normal to feel worried when facing a new situation, and recognising that this is a natural reaction may assist in learning to manage stress (Hughes et al., 2011). Thus, the Mind Tutor included micro-articles on each topic to highlight that it is normal to feel certain ways.

### Other Functions

**Fig. 2:**
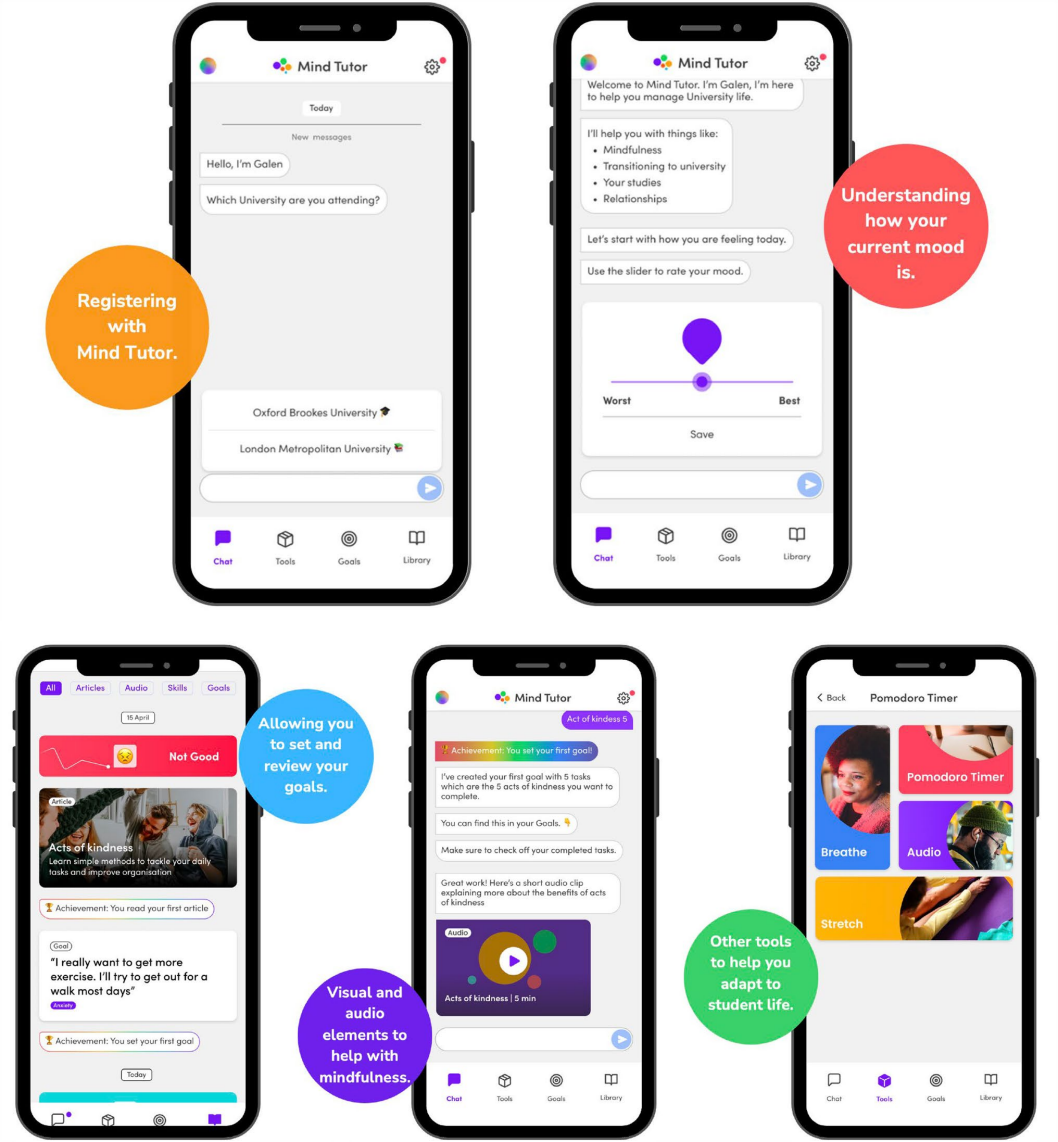
Screenshots of the Mind Tutor app

Mind Tutor (see Fig. 2) also included functionality to assist with recording and monitoring of individual goal-setting activities. It also had tools to assist with breathing exercises and focus timers specifically the incorporation of a ‘Pomodoro Timer’ (a tool to assist with focus by selecting a task and setting a timer to focus on that task). The breathing exercise app provided visual stimulation to assist with breathing exercises. Furthermore, there was a library functionality of content the student had engaged with while using the app, to allow them to revisit this content.

### App Development ‘Sprints’ and Description of Work by Software Company

The development of the Mind Tutor app was completed using an agile development methodology based on a series of 2-week ‘sprints’. Sprints are a short, time-boxed period when a team works to complete a set amount of work. The features to be developed in each sprint were taken from the sprint backlog (a clear list of tasks to be achieved in the sprint), which at the outset contained all the required functionality for the app. The backlog was subdivided into ‘epics’ (an epic is a large body of work that can be broken down into a number of smaller stories, or sometimes called ‘issues) which grouped the major work components. At the outset of each sprint, a ‘grooming’ (grooming is the practice of prioritising user stories in the product backlog to ensure they are ready for sprint planning) exercise was completed to determine which functionality would be developed within the sprint. At the end of the 2-week sprint, the developed features would be demonstrated and sign-off given by the product owner. This process continued to iterate until all the required features were delivered.

Each sprint consisted of members of the user experience (UX) team responsible for designing the user experience with the app, the integration team responsible for completing integration to systems including Moodle and the interaction design team responsible for implementing the user interaction flows. When work within the sprint was signed off by the product owner, the functionality was released to the quality assurance team who undertook usability and integration testing as necessary. On the completion of the functionality within an epic, a ‘show and tell’ session was held with the wider project research team and key stakeholders.

The Mind Tutor app was built utilising the pre-existing Syndeo AI platform. At its core, the platform facilitates the design and build of chatbot functionality. Interaction flows can be created and inputs captured from end-users. User inputs are captured in multiple ways—free-form text or selecting options using graphical elements such as carousels or reply buttons. The Syndeo AI Conversational AI platform facilitates the design and construction of these interaction flows as well as the interpreting of the user responses using natural language processing (NLP). Based on the interpretation of the user input, the platform then determines the next best action on dealing with that user prompt. The Mind Tutor app is therefore a multiplatform (covering Android and iOS) mobile app which can communicate with the Syndeo AI Conversational AI platform. The app was built using the Flutter framework (an open-source framework for developing an app’s user interface).

Within the Mind Tutor app, students interact with a chatbot, which identifies which of the five listed topics they need help with. This is based on a data model and an algorithm which accepts the user input (any user interaction) which is defined as an ‘intent’, the algorithm is used to ‘understand’ this intent (the intent could be free text or the user selecting a visual prompt) based on the data trained in the data model. The intent is then mapped to one of the five listed topics. Once this is identified, the Mind Tutor directs the participant to receive one of the five interventions. Participants may complete only one or all possible interventions within that topic. They may also go back to the main menu and start looking at another topic. The Mind Tutor app was also integrated into the learning platform (Moodle) of the students. This allowed the app to push out notifications to the students once new teaching materials were posted on the students’ learning platform. This integration aimed to facilitate the perception of students that the app is directly linked to their learning at university. Integration was developed with Moodle using exposed Application Programming Interfaces (APIs). The Mind Tutor app notifies the user based on the number of ‘unseen activities’ since they last accessed their course on Moodle. See Fig. 2 for screenshots of the app.

### Aim 2: Acceptability of and Engagement with Mind Tutor

#### Acceptability of Content

The Mind Tutor group were asked how useful they had found each topic in the app, and the control group were asked how useful they would find such topics if they were to have used the app. The most useful content for the app group was about transitions (*M* = 3.84, SD = 1.63), and the least useful was about relationships (*M* = 3.49, SD = 1.70). The control group rated academic content as potentially the most useful (*M* = 4.02, SD = 1.00), and relationship content the least potentially useful (*M* = 3.36, SD = 1.2.5—see Table 4). Suggestions for additional content from the Mind Tutor group included making friends, physical health, academic referencing, breaking bad habits, academic writing, coping with a poor grade, nutrition, getting active and contact lists for sources of support.

**Table 4:** Acceptability of content ratings for study participants

	Academic	Relationships	Mood	Transitions	Worry
Mind tutor *N* = 80					
*M* (SD)	3.60 (1.41)	3.49 (1.70)	3.63 (1.59)	3.84 (1.63)	3.69 (1.57)
Control–potential acceptability *N* = 125					
*M* (SD)	4.02 (1.00)	3.36 (1.25)	3.98 (1.07)	3.57 (1.15)	3.84 (1.17)

Participants in the control group made suggestions including mindfulness, calming techniques, timesheet for study, stress and worries, mental health problems, reassurance, study goals, how to keep focus and how to help other people in distress.

#### Engagement with Mind Tutor

Eighty-one participants provided comments about what would encourage students to use the app more often. The majority of comments (22) around app usage were around the notion of building in rewards, incentives and reminders. Thirteen were around the lack of interactivity and the lack of engaging content presentation. This was followed by 10 comments around lack of user friendliness, not quite understanding how the app works and too cumbersome to use. Eight comments were around the notion of presentation of content such as more videos or specific tasks to complete.

A number of comments were made about the chatbot. Positive comments included:

*It did not engage me as much as I had hoped, but the idea is really good (man, age 25).*

*Loved the app, still needs a lil tweaking but overall it’s amazing and so useful (woman, age 19).*

Negative comments included:

*Being able to stop the chat whenever I wanted and not having to complete the entire discussion (woman, age 19).*

*Took too long to find any solutions cos you had to talk the robot into it (man, age 19).*

Finally, participants were asked, ‘*What else would have helped you in the last few weeks’.* Responses to this question provide some indication of the range of other challenges that students were facing. A number of comments referred to the need to talk to someone about issues, for example:

*Probably talking with someone who could have helped me in seeing things more clearly and from different perspectives (Woman, age 19).*

Other students made broader points about coping with daily life, for example:

*Personally i think it’s just a matter of myself, like I could have done things that an app wouldn’t be able to do, like cleaning my room more often or going on walks. I don’t really think the app could do anything more except maybe ask about what i’ve done today and encourage me to do things like that? But i feel like that’s kind of a tall order for an app (Woman, age 18).*

#### App Usage

Across both trials, 85 participants provided self-report data about their use of the Mind Tutor. Of those, 10 (11.8%) reported never using the app, 15 (17.6%) reported using it once or twice, 22 (17.6%) said three or four times, 8 (9.4%) said once a month, 15 (17.6%) said once a week, 14 (16.5%) used the app a few times a week, and one person (1.2%) said they had used it every day. Five participants gave an answer as to why they did not download the Mind Tutor. One said that it was in case they had to pay for something, one did not want to and the others had not had the time.

#### Engagement Data from the Software Company.

There were 60 unique app users in the first trial, producing 114 interactions with the app. An interaction was defined as a continuous engagement with the chatbot. Of those, 53% only had one single interaction with the app, whereas 42% had between 2 and 4 interactions and the remaining 5% had more than four interactions. There were 84 unique app users during the second trial and 187 interactions. Engagement in the first 14 days was reasonably high with an average of 9.7 interactions a day. The engagement data from an individual user perspective were slightly better for trial 2; 56% only had one single interaction with the app, 26% had between 2 and 4 interactions, but 18% reported more than four interactions with the app. Overall, for both trials, engagement peaked in the first 2 weeks and then dropped off towards the end of the trial. During the trials on average, each student interacted with the app an average of two times.

## Discussion

This paper described the development process of an AI enhanced app for student wellbeing, the Mind Tutor, assessed its acceptability and explored engagement with the app. The development process involved engagement with a range of stakeholders, including students and support services. During development work, the findings suggested that managing workload was a significant challenge, alongside developing new relationships and support networks and addressing mental health challenges. This development work led to the selection of five key topics for the Mind Tutor to focus on: anxiety, mood, management of academic work, transition and relationships.

To our knowledge, this was the first chatbot app to incorporate undergraduate academic and life goals with mindfulness and psychoeducation. Ponzo et al. (2020) found an app-based programme including psychoeducation, mood tracking and deep breathing relaxation practices that improved UK University students’ wellbeing and anxiety over 4 weeks, but in a group who met the criteria for baseline depression, anxiety and stress, rather than the current study’s recruitment from whole year groups. Walsh et al. (2019) small randomised controlled trial found small improvements in Canadian undergraduate mood, stress and attention control over 3 weeks, but this was a mindfulness-only app. Thus, the insights from the development of the Mind Tutor have relevance for those developing and testing apps that combine a range of features and are aimed at the whole student body.

The strengths of the focus group study were that a diverse range of students took part and that they generated a range of viewpoints on important topics. However, it was limited in that a small number of students took part, and questions were preplanned and focused on academic skills and wellbeing, rather than allowing a natural discussion to take place about the challenges of student life. Focus groups can be viewed as a consensus method, but are subject to social desirability (Kitzinger, 1995).

Data from the host institution’s wellbeing and student support services were in line with the wider literature in suggesting that anxiety and depression are common issues for students (Frampton & Smithies, 2021; Ibrahim et al., 2013). Financial issues were also common, although the app did not offer this topic, due to constraints of focusing on five most common areas. Gaining feedback in a lecture from students was useful in corroborating the other data sources and selecting the five key areas. In addition, the students rated ‘activities to improve focus’ as highly important, leading to the creation of bespoke mindfulness activities in Mind Tutor. However, this feedback was opportunistic and only gained from students who attended their lecture that day. Student attendance was poor on the module, in line with attendance in other modules at the host institution since the COVID-19 pandemic. Those students who did not attend the lecture may have been facing different stressors relating to their personal and academic life compared with those who attended.

We also aimed to explore the acceptability of and engagement with the Mind Tutor as an intervention to improve student wellbeing and attainment. Generally, aspects of the app could be considered to be acceptable to the target population. The topics were rated as potentially useful and relevant, and open-ended comments revealed that some students had positive experiences of the app. Transitions were rated as the most useful topic. This is an important finding because few other apps focus on this issue. It is also an important area for future research. The use of mindfulness and goal setting were also highly acceptable to students who had not experienced the app, suggesting that these features would be of use to a wider pool of students. Further work should explore how to deliver these types of interventions in an engaging way, because acceptability does not always lead to engagement or effectiveness (Ehrlich et al., 2023). Engagement data showed that students did not engage with the app very frequently, although a limitation is that we do now know how long each interaction was for those who did. Some students in the Mind Tutor group did not download the app at all. Where students did download the app, most usage was in the first 2 weeks of the trials. This suggests that more work needs to be done to keep students interested and engaged if the app is to be used longer term.

### Key Considerations and Recommendations for Future Research

A number of important lessons have emerged from this study, and our RCTs, which may be of use to other researchers in the field hoping to develop and test digital interventions for student wellbeing. One consideration for future research concerns the study design. While randomised controlled trials are viewed as the gold standard for assessing intervention effectiveness (Hariton & Locascio, 2018), it is often challenging to conduct high quality trials, with many online studies similarly suffering from lower than anticipated recruitment and retention rates (Hall et al., 2020). We allowed students to use the app on a voluntary basis and engagement with the app was low. An improved approach to understand more about acceptability could be to require a minimum level of interaction with Mind Tutor in the trial, although this may lead to further drop out and may not mimic app use in a non-trial context, and so provide little knowledge about its efficacy. Students also self-selected into the study, rather than being identified as having any particular support needs relating to the topics on offer. Another important point is that app was offered as a standalone solution and was not integrated into the wider curriculum (despite plans to do so). A stronger integration with learning content as well as a better integration with other wellbeing offers from the university might have increased engagement. Students have a range of new tools and systems to use at university, and an additional standalone app may not be the most optimal way to support them.

In order to improve acceptability and engagement, there are further key considerations. Although the development process was grounded in a qualitative understanding of the issues in line with good practice (Yardley et al., 2015), we used a consultation model, rather than a fully student driven co-production model (Smith et al., 2022). Including students as co-researchers may have generated additional important insights (Slay & Stephens, 2013), and co-produced interventions are often lead to better recruitment and retention within RCTs (Hall et al., 2020).

There are a range of individual differences in reasons for engaging with smartphone apps, such as how useful participants perceive the app to be, and perceived lack of time (Perski et al., 2019). A systematic review found that app engagement could relate to either the participant, or the app itself with mood and values having an influence. There were also design elements that could impact app engagement such as the types of rewards available, the duration of the study and level of contextualisation (Antezana et al., 2022).

Students may also be overwhelmed by the number of apps they use daily, preferring to seek support face to face. For example, in our follow-up questionnaires, students mentioned a need to talk to someone about their issues. Pretorius et al. (2019) found that using a mental health app was the least favourable option for mental health help seeking, with only 8% of students stating they would use this source. While initial triage and signposting may be appropriate for an app, it is important that students with urgent concerns can be seen face to face (Vilaza & McCashin, 2021).

Other research with students also demonstrate low retention rates. Conflicting schedules and time commitment are identified frequently as obstacles for participation (Massie et al., 2015), and students often engage in a cost/benefit analysis when considering research participation (Khatamian Far, 2018). Mind Tutor included a wide range of content for students to explore, and some studies have shown that as the level of commitment increases, the retention level decreases (Cyr et al., 2013). It was made clear to potential participants that their data would remain confidential and would be stored securely; however, they may have doubted this information, which may have influenced trial recruitment (Mercurio et al., 2020).

## Conclusions

Overall, we found that that the Mind Tutor app content could be considered acceptable, but engagement was poor. This paper has demonstrated significant points of benefit and learning for future research on student wellbeing relating to trial design, co-production, engagement and recruitment.

## Data Availability

The data that support this study are available from Oxford Brookes University’s institutional repository—RADAR at 10.24384/dgzp-wn97.
